# Supplementary figures and images for: Mind the gender gap: COVID-19 lockdown effects on gender differences in preprint submissions
Source: PLoS One. 2022 Mar 25;17(3):e0264265. doi: 10.1371/journal.pone.0264265 (PMC8956178; doi:10.1371/journal.pone.0264265)

## Random effect: category

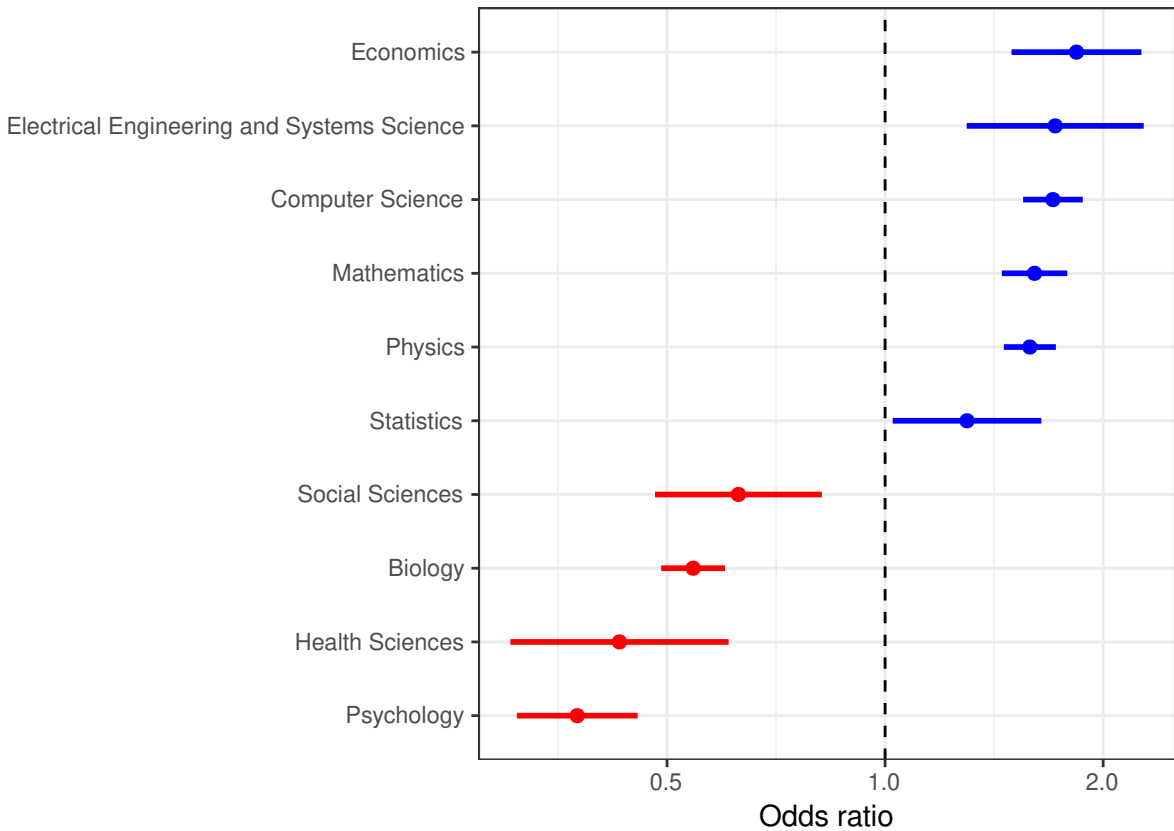

Supplement: S1 Fig — The model captures the known trends for all the major categories. Economics, engineering, computer science, mathematics, physics, and, to a lesser extent, statistics are categories with a proportion of males over the global average. By contrast, social sciences, biology, health sciences, and, specially, psychology are more balanced than the average. (PDF) [file pone.0264265.s001.pdf]

Random effect: subcategory

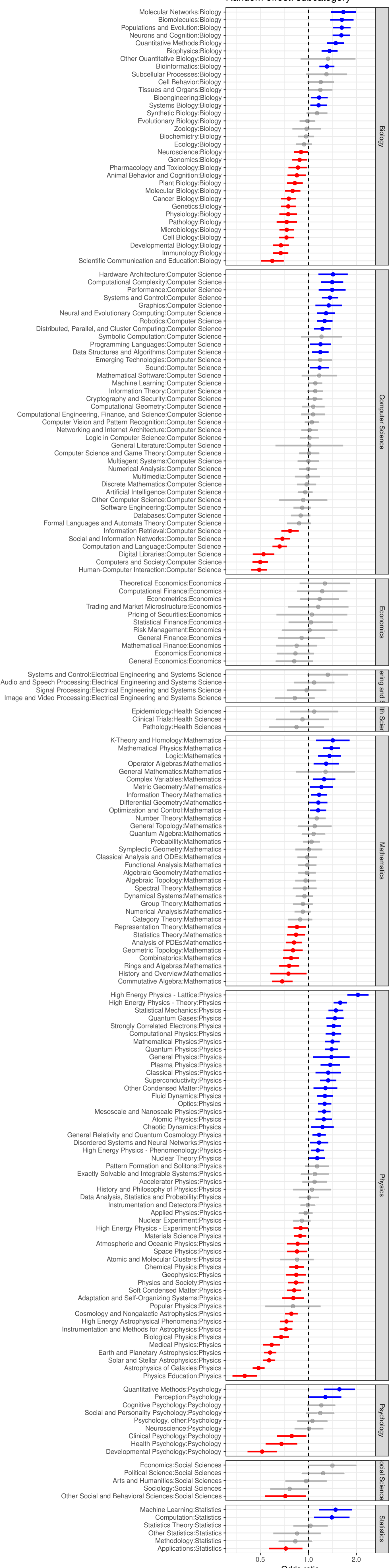

Supplement: S2 Fig — Within each category, specific subcategories develop their own trends. For example, we observe that high-energy and quantum physics are more masculinized than the average for all physics, while astrophysics-related research and bio-medical physics are more feminized. (PDF) [file pone.0264265.s002.pdf]
