# Supplementary material for: Mind the gender gap: COVID-19 lockdown effects on gender differences in preprint submissions
Source: PLoS One. 2022 Mar 25;17(3):e0264265. doi: 10.1371/journal.pone.0264265 (PMC8956178; doi:10.1371/journal.pone.0264265)
Supplement: S1 Table — Raw number of subcategories and preprints per category prior to any cleaning or pre-processing step. (PDF) [file pone.0264265.s003.pdf]

| Repository | Category | # subcategories | # preprints |
|---|---|---|---|
| arXiv | Computer Science | 40 | 113629 |
| | Economics | 3 | 1200 |
| | Elec. Eng. Systems Science | 4 | 11140 |
| | Mathematics | 32 | 104761 |
| | Physics | 51 | 184354 |
| | Quantitative Biology | 10 | 6186 |
| | Quantitative Finance | 9 | 2721 |
| | Statistics | 6 | 25307 |
| bioRxiv | Biology | 26 | 39181 |
| medRxiv | Health Sciences | 53 | 6122 |
| PsyArXiv | Psychology | 223 | 17515 |
| SocArXiv | Social Sciences | 419 | 10397 |
| Total: | | 876 | 522513 |
