# Supplementary material for: Mind the gender gap: COVID-19 lockdown effects on gender differences in preprint submissions
Source: PLoS One. 2022 Mar 25;17(3):e0264265. doi: 10.1371/journal.pone.0264265 (PMC8956178; doi:10.1371/journal.pone.0264265)
Supplement: S2 Table — As detailed in Methods, the large number of tags added to PsyArXiv and SocArXiv documents required a separate methodology for preprint categorization. As a final step of such methodology, SocArXiv also required manual recoding of the subcategories listed here. (PDF) [file pone.0264265.s004.pdf]

Subcategory	Recoded as
Science and Technology Studies	Other Social and Behavioral Sciences
Environmental Studies	Other Social and Behavioral Sciences
International and Area Studies	Other Social and Behavioral Sciences
Leisure Studies	Other Social and Behavioral Sciences
Organization Development	Other Social and Behavioral Sciences
Leadership Studies	Other Social and Behavioral Sciences
Library and Information Science	Arts and Humanities
Linguistics	Arts and Humanities
Urban Studies and Planning	Sociology
Social Statistics	Sociology
Social Work	Law
Legal Studies	Law
Public Affairs, Public Policy and Public Administration	Political Science
Agricultural and Resource Economics	Economics
