# Supplementary material for: Mind the gender gap: COVID-19 lockdown effects on gender differences in preprint submissions
Source: PLoS One. 2022 Mar 25;17(3):e0264265. doi: 10.1371/journal.pone.0264265 (PMC8956178; doi:10.1371/journal.pone.0264265)
Supplement: Attachment — Submitted filename: response.pdf [file pone.0264265.s006.pdf]

Responses to the reviewers of PONE-D-21-25133: “Mind the gender gap: COVID-19 lockdown effects on gender differences in preprint submissions”

Iñaki Ucar, Margarita Torre, Antonio Elías

February 5, 2022

We would like to thank the reviewers again for their time and valuable comments, which have been a great help in improving the paper. According to the feedback from revision 1, we are required to address one last comment:

> Reviewer 1: The authors have provided detailed responses to reviewers’ comments and have made appropriate amendments. The only thing that was not clear to me in the revised format was whether the change in the number of preprints (changes in data presented in table 1) resulted in any change in the results of the regression analysis. I see they are the same as before while the number of preprints has changed. I just want the authors to ensure that there hasn’t been an oversight in relation to this and the results are not erroneous.

We confirm that the results of the regression analysis were correct and didn’t change. It was just a mistake in the counts provided in our first version of Table 1, but the models were fed with the correct data, and the results have been checked several times. Accordingly, we proceeded to submit this new revision with no changes.
